# Effects of starting hemodialysis with an arteriovenous fistula or central venous catheter compared with peritoneal dialysis: a retrospective cohort study

**DOI:** 10.1186/1471-2369-13-88

**Published:** 2012-08-23

**Authors:** Luis Coentrão, Carla Santos-Araújo, Claudia Dias, Ricardo Neto, Manuel Pestana

**Affiliations:** 1 Nephrology Research and Development Unit, Faculty of Medicine, University of Porto & São João Hospital Centre, Alameda Professor Hernani Monteiro, Porto, 4202-451, Portugal; 2 Department of Health Information and Decision Sciences, Faculty of Medicine, University of Porto, Alameda Professor Hernani Monteiro, Porto, 4202-451, Portugal

## Abstract

**Background:**

Although several studies have demonstrated early survival advantages with peritoneal dialysis (PD) over hemodialysis (HD), the reason for the excess mortality observed among incident HD patients remains to be established, to our knowledge. This study explores the relationship between mortality and dialysis modality, focusing on the role of HD vascular access type at the time of dialysis initiation.

**Methods:**

A retrospective cohort study was performed among local adult chronic kidney disease patients who consecutively initiated PD and HD with a tunneled cuffed venous catheter (HD-TCC) or a functional arteriovenous fistula (HD-AVF) in our institution in the year 2008. A total of 152 patients were included in the final analysis (HD-AVF, n = 59; HD-TCC, n = 51; PD, n = 42). All cause and dialysis access-related morbidity/mortality were evaluated at one year. Univariate and multivariate analysis were used to compare the survival of PD patients with those who initiated HD with an AVF or with a TCC.

**Results:**

Compared with PD patients, both HD-AVF and HD-TCC patients were more likely to be older (*p*<0.001) and to have a higher frequency of diabetes mellitus (*p* = 0.017) and cardiovascular disease (*p* = 0.020). Overall, HD-TCC patients were more likely to have clinical visits (*p* = 0.069), emergency room visits (*p*<0.001) and hospital admissions (*p*<0.001). At the end of follow-up, HD-TCC patients had a higher rate of dialysis access-related complications (1.53 *vs.* 0.93 *vs.* 0.64, per patient-year; *p*<0.001) and hospitalizations (0.47 *vs.* 0.07 *vs.* 0.14, per patient-year; *p* = 0.034) than HD-AVF and PD patients, respectively. The survival rates at one year were 96.6%, 74.5% and 97.6% for HD-AVF, HD-TCC and PD groups, respectively (*p*<0.001). In multivariate analysis, HD-TCC use at the time of dialysis initiation was the important factor associated with death (HR 16.128, 95%CI [1.431-181.778], *p* = 0.024).

**Conclusion:**

Our results suggest that HD vascular access type at the time of renal replacement therapy initiation is an important modifier of the relationship between dialysis modality and survival among incident dialysis patients.

## Background

Early referral of chronic kidney disease (CKD) patients to nephrology centres may enable patients to be adequately informed regarding the different renal replacement treatment (RRT) modalities [hemodialysis (HD), peritoneal dialysis (PD) and kidney transplantation (TX)], leading to better results in terms of morbidity and mortality [1-4]. Large registry-based studies have suggested a survival advantage of PD over HD, particularly during the first 1 to 2 years of treatment [5,6]. Although the ability of PD to provide better preservation of residual renal function was invoked as a possible explanation for the survival advantage of PD over HD during the first years of treatment, case mix differences in patients initiating HD may have confounded the interpretation of the studies that examined the influence of the dialysis modality on patient survival [5-7].

The type of vascular access used in HD patients is recognized to have a significant influence on survival. The use of a tunneled cuffed catheter (TCC) is associated with a substantially greater risk of sepsis, hospitalization and mortality compared to the use of an arteriovenous fistula (AVF) [8-12]. Although technique survival with PD is shorter than that with HD, in part due to access-related infections, the frequency of PD catheter-related complications has decreased in recent years, with a low rate of bacteremia/sepsis [13,14]. However, there are few studies comparing the outcomes of incident PD patients with those of HD patients using different vascular access types at dialysis initiation in the literature, to our knowledge [15,16]. In the study presented here, we hypothesize that vascular access type at the time of dialysis initiation accounts for the higher early mortality rate observed in patients who start HD with a catheter, compared to those who initiate HD with a functioning fistula or PD. To test our hypothesis, we compared all-cause and dialysis access-related morbidity/mortality between PD and HD patients with the latter stratified by HD vascular access type at dialysis initiation.

## Methods

### Study design

We conducted a retrospective observational cohort study among CKD patients (age 18 years and older at the start of RRT) who consecutively initiated HD between January 1 and July 1 2008, or PD between January 1, 2008 and July 1, 2009, in our institution.

The study was approved by the Ethics Committee for Health and the Local Institutional Review Board of São João Hospital Centre, Porto, Portugal.

### Setting

Portugal has a higher incidence of end stage-renal disease, ESRD (i.e. the patients who start any RRT modality for the first time) and prevalence in compared to most of other European countries. In 2009, an incidence rate of 240 and a prevalence of 1507 patients per million of the population were registered in ERA-EDTA [17]. Specifically, 10,152 patients underwent HD and 660 patients PD in 2010 (registered by the Portuguese Society of Nephrology). In Portugal, HD is almost exclusively (~90%) provided by outpatient hemodialysis units run by private providers. Hemodialysis patients undergo 4 hours of dialysis three times weekly, aiming for a spKt/V of 1.4 or greater. Patients undergo treatment using high-flux dialyzers; no hemodialyzer is reused. Peritoneal dialysis is provided by public hospitals and university centres. Patients attending our hospital center undergo either continuous ambulatory peritoneal dialysis (CAPD) or automated cyclic peritoneal dialysis (CCPD). All patients have a 1 to 2 week training period before initiation of therapy at home. Treatment of PD patients is individualized: the total Kt/V (renal and peritoneal clearance) aimed for is 1.8 or more and the majority of patients are treated with dextrose-based solutions with daily exchange with Extraneal (Baxter Healthcare Corp, Deerfield, IL, USA).

### Patients

The patients were recruited from the Department of Nephrology of São João Hospital Centre which is a tertiary-care University Hospital responsible for nephrological medical support to ESRD patients beginning RRT within the northwest region of Portugal. Patients were enrolled if they had a diagnosis of end-stage CKD according to a nephrologist and had received outpatient chronic dialysis treatment. Patients who had previously undergone RRT (HD, PD or TX) and restarted during the study period and patients transferred to another district immediately after starting RRT were excluded. The RRT modality adopted was based on patient choice and his/her medical status. Initial dialysis modality was defined as the modality at the first outpatient dialysis treatment: patients starting PD therapy assigned to the PD group and patients starting HD therapy with a tunnelled cuffed catheter or a functioning fistula to the HD-TCC or HD-AVF groups, respectively. Although changes in vascular access type were recorded during follow-up, patients remained in the same index group. Follow-up started on the day dialysis was first performed as an outpatient and continued for 1 year or until death or switching from the RRT modality. Because of the relatively lower number of patients who initiated PD between January 1, 2008 and July 1, 2008 compared to those who initiated HD, the recruitment period for incident PD patients was extended to July 2009.

A total of 191 CKD patients started RRT during the study period (133 HD, 58 PD). Twenty-three HD patients were excluded from the study due to previous RRT (n = 13) or loss to follow-up because of transfer to another district (n =10). In addition, 16 PD patients were excluded from the study because they had previously undergone RRT (HD, 11 patients; TX, 5 patients). A total of 152 patients were included in the final analysis. Of the 110 incident HD patients, 59 started therapy with a functioning AVF and 51 with a TCC. Three cohorts of incident dialysis patients were then established: HD-AVF (n = 59), HD-CVC (n = 51) and PD (n = 42).

### Data

Clinical data and information regarding access type were collected from our hospital database and from outpatient dialysis unit records, when appropriate. A physician assessed the presence of co-morbid illness by complete review of each patient’s records at the enrolment date. Information was collected for the 19 variables that constitute the Charlson Comorbidity Index [18], which has been validated for use in patients with ESRD. The number of clinical and emergency room visits, hospitalizations and dialysis access complications were determined for all participants from our hospital database and from outpatient dialysis unit records, when appropriate.

Complications of HD and PD accesses were classified as mechanical or infectious events [19,20]. Mechanical complications included AVF stenosis, thrombosis, bleeding and limb ischemia; TCC flow dysfunction, thrombosis, bleeding, cuff extrusion and complications of central venous catheterization; PD catheter flow dysfunction, bleeding, leaks, cuff extrusion, hernias and complications related to Tenckhoff catheter placement. Infectious complications included AVF-related bacteremia, TCC-related bacteremia, PD-related peritonitis and bacteremia.

Dates of renal transplantation, switch from the RRT modality and/or death were known until end off follow-up.

### Outcomes

The primary aim of this analysis was to determine the all-cause mortality of HD-AVF, HD-TCC and PD patients at 1 year from the time of first dialysis.

A secondary aim was to examine the dialysis access-related morbidity/mortality of HD-AVF, HD-TCC and PD patients at 1 year from the time of first dialysis.

### Statistical analysis

Data are given as percentages and means ± SD. Categorical variables were compared using Fisher’s exact test. The Kruskal-Wallis test was used to analyze differences between continuous variables. Rates were calculated for each patient by dividing the number of events/procedures by the duration of follow-up in years. Survival on dialysis was calculated by the Kaplan-Meier method. Univariate analysis of survival was performed by the log rank method. Multivariate analysis of survival was performed using a Cox proportional hazards model. Covariates were included if the baseline difference between the three groups was <0.10. All tests were two sided, and differences were considered significant at *P*<0.05. All statistical analyses were performed using the SPSS software, version 19 (SPSS, Inc., Chicago, IL, USA).

## Results

### Baseline characteristics

Table 1 lists the baseline characteristics of the study population. Compared with PD patients, both HD-TCC and HD-AVF patients were more likely to be older (*p*<0.001, Table 1) and to have a higher frequency of diabetes mellitus (*p* = 0.017, Table 1), coronary heart disease (*p* = 0.007, Table 1) and congestive heart failure (*p* = 0.023, Table 1). Both HD-AVF and PD groups initiated dialysis with similar levels of serum hemoglobin and serum albumin. In addition, ~80% of both HD-AVF and PD groups were referred to a nephrologist early. HD-TCC patients were more likely to be referred to a nephrologist late (*p*<0.001, Table 1), and to initiate dialysis with lower hemoglobin (*p*<0.001, Table 1) and serum albumin (*p*<0.001, Table 1). HD-AVF patients were more likely to initiate RRT with higher estimated glomerular filtration rate (eGFR) than either HD-TCC or PD patients (*p*<0.001, Table 1).

**Table 1:** Baseline characteristics of enrolled patients treated with different dialysis modalities and vascular accesses (HD-AVF, hemodialysis with arteriovenous fistula; HD-TCC, hemodialysis with catheter; PD, peritoneal dialysis)

**Variable**	**HD-AVF (n = 59)**	**HD-TCC (n = 51)**	**PD (n = 42)**	**P**
Male sex (%)	60%	55%	52%	0.856
Mean age (y)	62.8 ± 14.3	66.1 ± 15.4	55.1 ± 16.1	0.001
*18-44 years*	5 (9%)	4 (8%)	9 (21%)	0.047
*45-64 years*	19 (32%)	12 (24%)	20 (47%)	0.015
*65+ years*	35 (59%)	35 (69%)	13 (31%)	0.001
Etiology of kidney disease (%)				
*Diabetes*	26 (44%)	22 (42%)	8 (19%)	0.017
*Hypertension*	7 (12%)	4 (8%)	2 (5%)	0.471
*Glomerulonephritis*	7 (12%)	3 (6%)	13 (31%)	0.003
*Tubulointersticial kidney disease*	8 (14%)	10 (20%)	7 (17%)	0.702
*Unknown*	11 (18%)	12 (24%)	12 (29%)	0.510
Mean Charlson Comorbidity Index	5.1 ± 3.1	5.0 ± 2.5	4.4 ± 2.2	0.574
*Low risk (≤ 3)*	25 (42%)	17 (34%)	15 (36%)	0.745
*Medium risk (4–5)*	13 (22%)	11 (21%)	14 (33%)	0.133
*High risk (≥6)*	21 (36%)	23 (45%)	13 (31%)	0.575
Comorbid conditions (%)				
*Coronary heart disease*	26 (44%)	17 (33%)	6 (14%)	0.007
*Congestive heart failure*	25 (42%)	18 (35%)	7 (17%)	0.023
*Peripheral vascular disease*	14 (24%)	11 (22%)	9 (19%)	0.104
*Previous stroke*	7 (12%)	8 (16%)	2 (5%)	0.095
*Diabetes*	26 (44%)	23 (45%)	8 (19%)	0.015
*Malignant disease*	10 (20%)	10 (23%)	11 (26%)	0.432
Late referral (%)	13 (22%)	44 (86%)	9 (21%)	<0.001
Time from referral to dialysis initiation, months (mean ± SD)	39 ± 35	11 ± 30	34 ± 28	<0.001
Hemoglobin (g/L)	104 (101, 108)	90 (85, 94)	105 (108, 115)	<0.001
eGFR (ml/min per 1.73 m^2^)*	10.0 (9.2, 10.9)	7.8 (6.8, 8.9)	8.3 (7.7, 9.0)	<0.001
Serum creatinine (mg/dL)	5.7 (5.3, 6.1)	8.0 (7.0, 9.1)	6.7 (6.0, 7.4)	<0.001
Serum urea (mg/dL)	218 (203, 231)	217 (194, 239)	197 (184, 210)	0.214
Serum albumin (g/L)	37 (35, 38)	33 (31, 34)	39 (38, 40)	<0.001

* eGFR, estimated glomerular filtration rate.

### Patient outcomes

Table 2 lists the mean numbers of clinical events of the study population.

**Table 2:** Dialysis access-related and overall clinical events of enrolled patients treated with different dialysis modalities and vascular accesses (HD-AVF, hemodialysis with arteriovenous fistula; HD-TCC, hemodialysis with catheter; PD, peritoneal dialysis), per patient-year at risk (mean ± SD)

**Clinical events**	**HD-AVF**	**HD-TCC**	**PD**	**P**
	**(n = 59)**	**(n = 51)**	**(n = 42)**	
**Dialysis access-related**				
Mechanical complications	0.93 ± 1.40	0.82 ± 1.49	0.07 ± 0.26	<0.001
* Fistula related*	0.73 ± 0.99	0.29 ± 0.64	0	<0.001
* Catheter related*	0.20 ± 0.71	0.53 ± 1.12	0.07 ± 0.26	0.114
Infectious complications				
*Patients infection free, at year 1, N (%)*	59 (100%)	33 (65%)	24 (57%)	<0.001
* Peritonitis*	0	0	0.57 ± 0.74	0.002
* Bacteremia*	0	0.71 ± 1.29	0	0.004
Total	0.93 ± 1.40	1.53 ± 1.89	0.64 ± 0.83	<0.001
**Overall**				
Dialysis access-related complications *	0.93 ± 1.40	1.53 ± 1.89	0.64 ± 0.83	<0.001
Clinical visits	4.17 ± 4.29	6.35 ± 10.25	3.38 ± 3.41	0.069
Emergency room visits	1.42 ± 2.38	3.06 ± 3.23	1.62 ± 1.75	<0.001
Hospital admissions	0.66 ± 1.14	2.04 ± 1.55	0.50 ± 0.74	<0.001
* Dialysis accesss-related*	0.07 ± 0.25	0.47 ± 1.09	0.14 ± 0.42	0.034
* Other*	0.59 ± 1.03	1.57 ± 1.05	0.36 ± 0.62	0.010
Total	7.18 ± 6.76	12.98 ± 12.61	6.14 ± 4.12	<0.001

* Includes all dialysis access-related mechanical and infectious complications.

HD-TCC patients were more likely to have higher numbers of dialysis access-related complications than HD-AVF and PD patients (*p*<0.001, Table 2). In particular, the PD group had the lowest number of mechanical access-related complications (*p*<0.001, Table 2) and the HD-AVF group the lowest infection rate (*p*<0.001, Table 2). Despite the similar number of infection-free patients in the PD and HD-TCC groups at 1 year of follow-up, both catheter-related bacteremia and hospital admissions were significantly higher in the HD-TCC group (*p* = 0.004 and 0.034, respectively; Table 2).

Overall, HD-TCC patients were more likely to have clinical visits (*p* = 0.069, Table 2), emergency room visits (*p*<0.001, Table 2) and hospital admissions (*p*<0.001, Table 2). The mean numbers of hospital days for HD-AVF, HD-TCC and PD patients were 5.5 ± 13.7, 36.6 ± 40.7 and 5.1 ± 15.1 days, per patient-year at risk, respectively (*p*<0.001).

**Figure 1:**
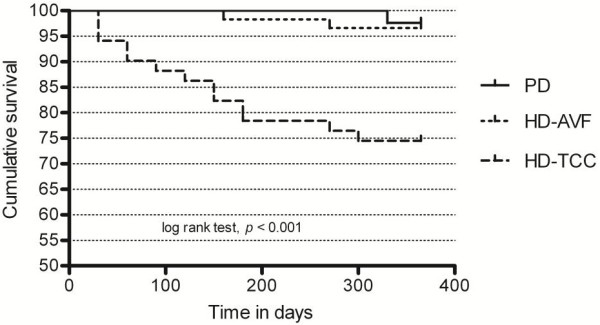
**Kaplan-Meier plots of survival in incident dialysis patients with log rank analysis to assess the significance of dialysis access on survival.** Survival curves for HD-AVF (hemodialysis with arteriovenous fistula, dotted line), HD-TCC (hmodialysis with tunneled cuffed catheter, dashed line), and PD (peritoneal dialysis, solid line) demonstrate higher 1-year mortality in HD-TCC patients.

Sixteen patients died during follow-up (HD-AVF, n = 2; HD-TCC, n = 13; PD, n = 1). The main causes of death for HD-TCC patients were catheter-related bacteremia (n = 7), cardiac disease (n = 4), pneumonia (n = 1) and cancer (n = 1); for HD-AVF patients was cancer (n = 2) and for PD patients was pyonephrosis (n = 1). The survival rates at one year were 86.3% and 97.6% for HD and PD patients, respectively (*p* = 0.044, log rank test). When stratified for HD vascular access type, the survival rates at one year were 96.6%, 74.5% and 97.6% for HD-AVF, HD-TCC and PD groups, respectively (Figure 1; *p*<0.001, log rank test). Older age (*p* = 0.002), diabetes (*p* = 0.006), cardiovascular disease (*p* = 0.026), late referral (*p* = 0.001) hypoalbuminemia (*p* =0.001) and anemia (*p* =0.002) were all associated with poorer survival by log rank analysis. The impact of HD vascular access at the time of dialysis initiation on survival was considered in more detail in a multivariate model to correct for confounding variables. The results of the Cox model are given in Table 3- HD-TCC use at the time of dialysis initiation was independently associated with death (HR 16.128, 95%CI [1.431-181.778], *p* = 0.024).

**Table 3:** Results of the Cox multivariate analysis for the relationship between co-morbid factors, dialysis access at dialysis initiation and death in incident dialysis patients (HD-AVF, hemodialysis arteriovenous fistula; HD-TCC, hemodialysis tunneled cuffed catheter; PD, peritoneal dialysis catheter)

	**Hazard ratio**	**95% confidence intervals**	***P***
Age (per year)	1.080	0.996-1.171	0.062
Diabetes	0.487	0.139-2.288	0.318
Coronary heart disease	1.875	0.381-9.227	0.439
Congestive heart failure	0.497	0.117-2.158	0.497
Peripheral vascular disease	0.499	0.114-2.190	0.357
Previous stroke	0.197	0.032-1.225	0.081
Late referral	1.009	0.990-1.028	0.378
Albumin	0.917	0.814-1.033	0.153
Hemoglobin	0.999	0.948-1.054	0.975
eGFR*	1.135	0.903-1.426	0.279
Dialysis access			
* PD (reference)*			
* HD-AVF*	0.734	0.056-9.656	0.814
* HD-TCC*	16.128	1.431-181.778	0.024

* eGFR, estimated glomerular filtration rate.

At the end of follow-up, 97% (n = 57) and 47% (n = 18) of HD-AVF and HD-TCC patients had a functional fistula as permanent vascular access, respectively. Three patients switched definitely from PD to HD due to PD-related peritonitis (n = 2) and tuberculous peritonitis (n = 1). Only 2 patients received a transplant during the study period.

## Discussion

The study presented here shows that incident HD-TCC patients experienced a significantly higher mortality rate at one year of dialysis, in comparison with HD-AVF and PD patients. Infection was the most common cause of death, whereas the second most common cause was death related to cardiovascular disease. Dialysis access-related complications were responsible for 43% (n = 7) of all deaths, and infection was the single cause responsible for such deaths. Death caused by dialysis access complications occurred only in the HD-TCC group. Importantly, HD-TCC patients had approximately twice as many clinical events related to dialysis access than either HD-AVF or PD patients (mainly access-related bacteremia episodes and hospitalizations). In contrast, most of the vascular and peritoneal dialysis access complications in the HD-AVF and PD groups were not serious clinical events, and no dialysis access-related deaths occurred in either these two groups. Although HD-TCC patients had similar baseline characteristics to HD-AVF patients, HD-TCC patients were referred to the nephrologist later, which might explain the delay in AVF creation in this group. In contrast, both incident HD-AVF and PD patients were referred to the nephrologist early and could thus benefit from appropriate vascular and peritoneal access placement in due time. Despite different baseline characteristics, both the HD-AVF and PD groups had similarly high survival rates at year 1. Multivariate analysis showed that HD-TCC use at the time of dialysis initiation was the important factor associated with poor prognosis. Taken together, our results strongly suggest that HD vascular access type at the time of dialysis initiation might explain the differences in outcome observed between the incident HD and PD populations. Our results corroborate the recent findings of Perl *et al.*, [15] in incident adult dialysis patients on the Canadian Organ Replacement Register who found that patients initiating HD with a catheter had a higher risk of death compared to both HD-AVF and PD patients.

Our findings are also in agreement with the recent report of Quinn *et al.*, [21] that showed no difference in survival between PD and HD patients who received > 4 months of predialysis care. Also, Raithatha *et al.*[16] recently showed that the use of HD-catheter is one of the key features of late referral that determines poor prognosis. In the present study, ~80% of both HD-AVF and PD patients were referred to the nephologist early and experienced similarly high survival rates in the first year of dialysis, compared to HD-TCC patients. Our results support the need for early referral of ESRD patients to nephrology centers to provide the opportunity for patient selection of RRT modality and timely creation of the appropriate dialysis access [22].

Most reports that have used USRDS data do not include the critical initial 90-day period on dialysis. This is a time period when a high proportion of HD patients are using catheters as bridging access devices [12]. In the present study, survival rates of HD-TCC, HD-AVF and PD groups at 90 days of follow-up were 88%, 100% and 100%, respectively. Exclusion of this period in the analysis would probably underestimate the morbidity and mortality rates of the HD-TCC group.

One interesting finding of the present study was that bacteremia only occurred in HD-TCC patients, refuting the common misconception that PD is associated with an overall higher rate of severe infection than HD. In addition, PD patients had the lowest number of mechanical access-related complications. Our results support the previous findings of Oliver *et al.*[23,24] and Povlsen *et al.*[2,25,26] by showing that patients who choose PD require fewer access interventions and do not face an increased risk of access-related complications compared to HD patients.

As a retrospective study, this study has the limitations of such an approach. As with all observational studies, there may have been selection bias, in particular influenced by patient treatment preferences and time of referral to the nephrologist. PD patients were younger and had lower comorbid illness, compared to HD patients. The patient population consisted mainly of Caucasian Europeans, which makes it impossible to draw conclusions for other ethnic groups. Peritoneal dialysis patients were treated in a single academic nephrology centre, whereas HD patients were treated in separate peripheral renal centers, although this is a reflection of the local distribution of patients between modalities.

## Conclusion

Our study provides evidence favoring the view that HD vascular access type at renal replacement therapy initiation is an important modifier of the relationship between dialysis modality and survival among incident dialysis patients. Our results emphasize the need for an early referral program for ESRD patients so that those who choose HD have a functioning AVF, and those who choose PD have a Tenckhoff catheter placed in due time. We believe such a policy would decrease the risk of dialysis morbidity/mortality.

## Competing interests

The authors have no equity interest or financial agreements with any company or commercial entity related to the content of the article and they have not received salary or support from any company related to the article.

## Authors’ contributions

LC: participated in the design of the study, in the acquisition and interpretation of data, performed the statistical analysis and wrote the manuscript. CSA: participated in the acquisition and interpretation of data and revised the manuscript for important intellectual content. CCD: performed the statistical analysis. RN participated in the acquisition of data. MP: participated in the interpretation of data and revised the manuscript for important intellectual content. All authors read and approved the final manuscript.

## Pre-publication history

The pre-publication history for this paper can be accessed here:

http://www.biomedcentral.com/1471-2369/13/88/prepub
